# A CT radiomics nomogram predicts visual acuity improvement in patients with indirect traumatic optic neuropathy following optic canal decompression

**DOI:** 10.3389/fneur.2026.1819666

**Published:** 2026-05-18

**Authors:** Guangyu Wang, Pengran Yu, Shuo Li, Wenchuan Zhang

**Affiliations:** Department of Neurosurgery, Shanghai Ninth People's Hospital, Shanghai Jiao Tong University, School of Medicine, Shanghai, China

**Keywords:** clinical machine learning, nomogram, optic canal decompression, radiomics, traumatic optic neuropathy

## Abstract

**Introduction:**

Indirect traumatic optic neuropathy (ITON) is a common cause of persistent visual impairment following craniofacial trauma. While optic canal decompression (OCD) serves as a primary surgical intervention, its efficacy is variable, with approximately half of the patients failing to achieve significant visual acuity (VA) recovery. Existing models for predicting surgical outcomes lack sufficient accuracy and generalizability.

**Materials and methods:**

This retrospective, single-center study included 169 patients with ITON who underwent OCD between May 2014 and October 2025. Participants were randomly assigned to training (*n* = 118) and test (*n* = 51) sets in a 7:3 ratio. Radiomics features were extracted from preoperative CT scans, and the most prognostically significant features were identified via Least Absolute Shrinkage and Selection Operator (LASSO) regression. The model’s performance and clinical utility were evaluated through receiver operating characteristic (ROC) analysis, calibration curves, and decision curve analysis (DCA).

**Results:**

The final nomogram incorporated nine radiomics features and three clinical predictors. It demonstrated robust discriminative ability, with area under the curve values of 0.840 in the training set and 0.832 in the test set. This tool effectively categorized patients into distinct risk groups for poor VA recovery. VA improvement rates were significantly higher in the low-risk group than in the high-risk group in both the training (87.9% vs. 30.5%) and test (87.0% vs. 28.6%) cohorts. Calibration and decision curve analyses confirmed the superior performance and clinical net benefit of the combined model over those relying solely on clinical or radiomics features.

**Conclusion:**

The developed clinical-radiomics nomogram represents a valuable non-invasive tool for the preoperative prediction of VA improvement following OCD in patients with ITON, showing significant potential to inform personalized treatment strategies.

## 1 Introduction

Traumatic optic neuropathy (TON), a frequent complication of craniofacial or closed-head trauma with an incidence of 0.5–8%, typically manifests as unilateral or bilateral vision loss of varying severity, potentially leading to complete blindness (1, 2). Despite receiving timely treatment, approximately 50% of patients continue to experience irreversible visual acuity (VA) impairment, significantly impacting their quality of life (3). Based on the injury mechanism, TON is categorized into direct and indirect (ITON) forms (4). While the exact pathophysiology of ITON remains incompletely elucidated, it is generally attributed to the transmission of traumatic force from impact sites to the optic nerve, resulting in axonal edema, microvascular compromise, and ultimately, retinal ganglion cell apoptosis (5, 6).

The management of ITON encompasses a spectrum of approaches, including conservative observation, high-dose corticosteroids, optic canal decompression (OCD), and combined regimens (4, 7–9). However, the lack of high-quality evidence complicates clinical decision-making, with no universally accepted consensus on the optimal treatment selection (8, 10). OCD, which can be performed via transcranial, endoscopic endonasal, or transorbital routes, aims to relieve mechanical compression on the optic nerve (11). Although the efficacy of these surgical approaches is comparable, patient outcomes demonstrate significant heterogeneity, with reported VA improvement rates ranging widely from 18.8 to 81.2% (7, 12–19). This variability highlights the critical need for reliable prognostic tools to identify patients who are unlikely to benefit from surgery, thereby preventing unnecessary invasive procedures.

To screen patients suitable for OCD, previous studies have assessed certain clinical characteristics associated with OCD outcome, including initial VA, age, and optic canal fractures (OCF) (20–25). Unfortunately, prediction models based solely on these factors exhibit limited stability and accuracy across independent studies (12, 26, 27). Radiomics, an emerging field involving the high-throughput extraction of quantitative features from medical images, offers a promising solution (28–30). It can capture quantitative imaging heterogeneity that may reflect underlying tissue alterations beyond conventional visual assessment. Although CT is routinely used preoperatively to evaluate bony anatomy and fractures, it traditionally overlooks the nuanced textural and intensity patterns within the optic nerve parenchyma. Therefore, we hypothesize that radiomics analysis of the entire optic nerve on CT can yield valuable prognostic biomarkers. By integrating these image-derived features with clinical predictors into a nomogram, this study aims to develop a robust tool for the preoperative risk stratification of ITON patients, thereby facilitating the selection of optimal candidates for surgical intervention.

## 2 Materials and methods

### 2.1 Patient selection

**Figure 1:**
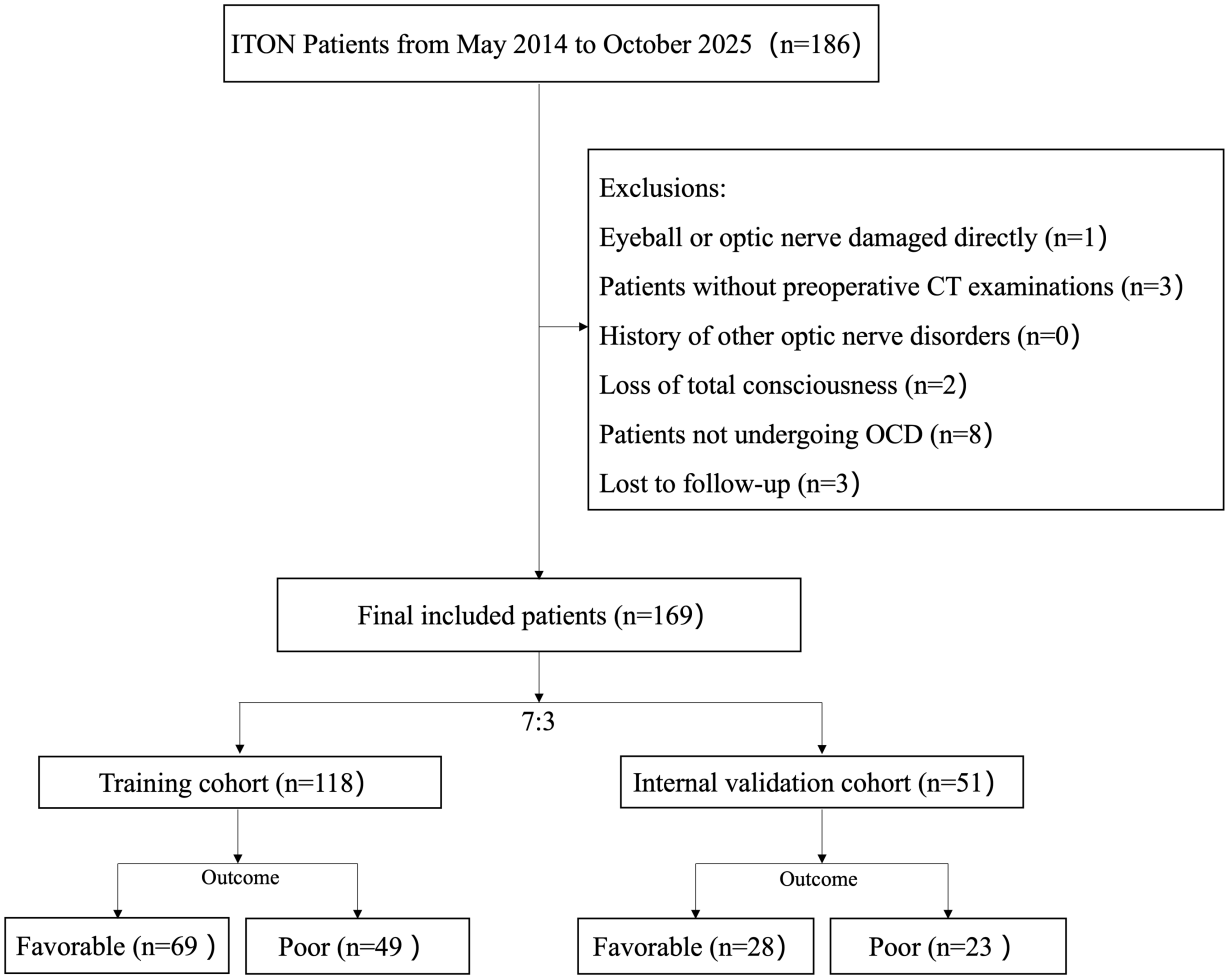
Enrolment and group assignment of study participants.

The institutional review board granted approval for this retrospective analysis and waived the need for informed consent. We initially identified 186 patients diagnosed with ITON between May 2014 and October 2025. Diagnosis relied on three key elements: (1) a documented history of craniofacial trauma; (2) observed visual impairment subsequent to the injury; and (3) the presence of a relative afferent pupillary defect. Eligible participants were conscious individuals with ITON who proceeded to OCD. We excluded patients who were comatose, did not receive surgical decompression, had incomplete CT imaging data, presented with pre-existing optic neuropathies, or were lost to follow-up before 3 months. Furthermore, patients with significant concomitant ocular injuries, such as globe rupture, vitreous hemorrhage, retinal detachment, or severe corneal opacification, were also strictly excluded to ensure that visual recovery was primarily attributable to OCD. Following the screening process, a total of 169 patients were included in the present study. The patient screening process is illustrated in Figure 1.

### 2.2 Treatment principles

All enrolled patients underwent OCD as the primary surgical treatment. The operative technique—whether pterional, supraorbital, or endoscopic transnasal—was tailored to each case, considering factors such as the pattern of associated skull base or facial fractures, the location of any intracranial hematomas, and the surgical team’s expertise. A common objective across all procedures was the removal of bone fragments impinging on the optic canal, aiming for a decompression arc of at least 180 degrees.

### 2.3 OCD outcome evaluation

Postoperative follow-up extended for a minimum of 3 months. Visual acuity was recorded and analyzed on a per-eye basis; laterality referred to the affected eye with ITON that underwent OCD, and the fellow eye was not averaged into the analysis. VA was evaluated preoperatively and at the latest follow-up. We classified VA into a five-tiered scale: (1) no light perception (NLP); (2) light perception (LP); (3) hand motion (HM); (4) finger counting (FC); and (5) measurable acuity on a standard chart. A favorable outcome was defined as an improvement of one or more levels from the preoperative baseline. This criterion for defining surgical success is consistent with established clinical standards in previously published literature on traumatic optic neuropathy (18, 31, 32). Absence of any change in VA level was classified as a poor outcome.

### 2.4 Image processing

**Figure 2:**
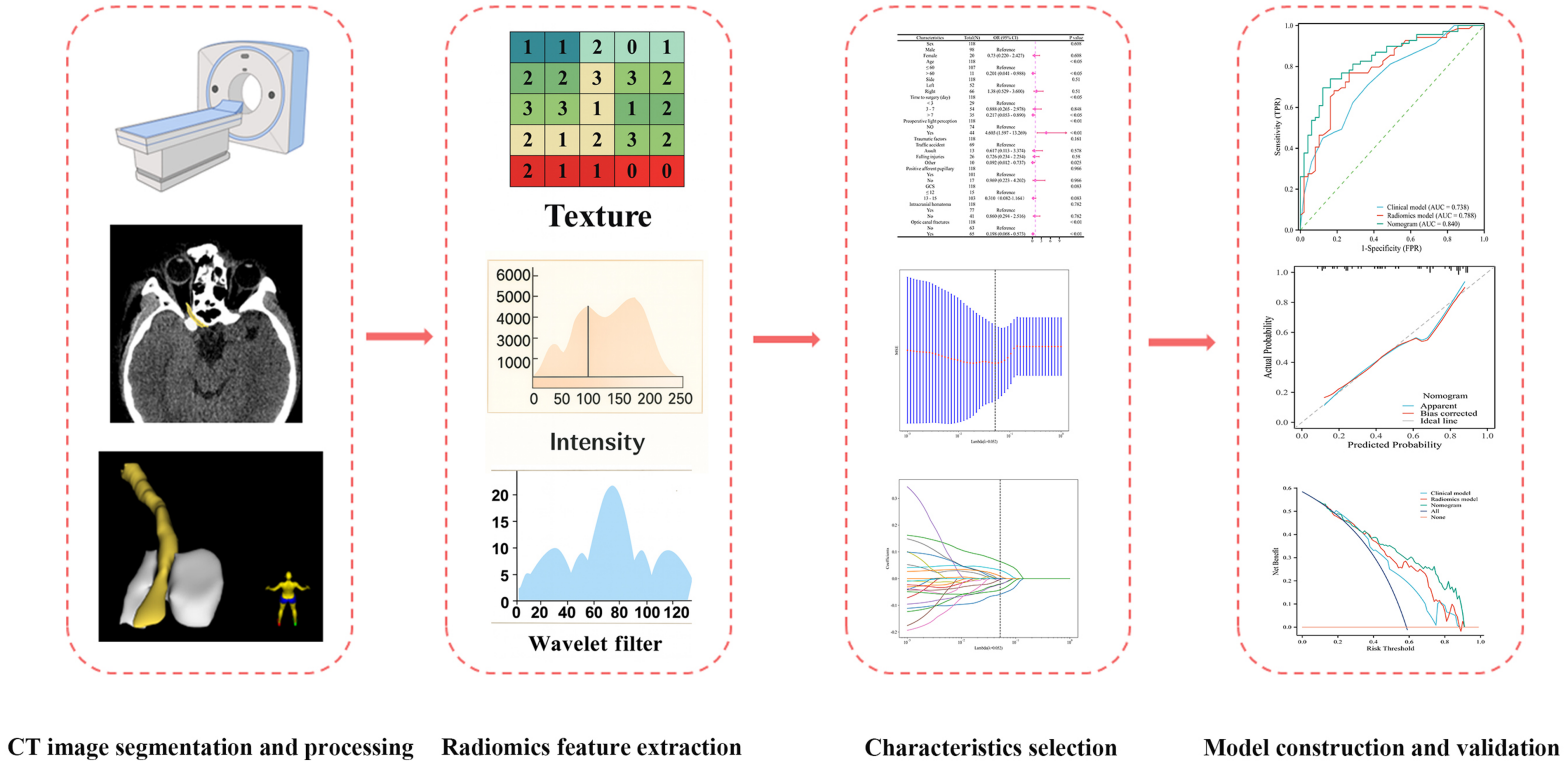
Schematic overview of the radiomics analysis workflow.

The workflow of this radiomics study is depicted in Figure 2. Prior to OCD, all patients underwent high-resolution non-contrast cranial CT scans with contiguous slices of 0.625 mm within 1 day after head trauma. These sequences are widely accepted for identifying optic canal fracture and hematoma. Before segmentation and feature extraction, the CT datasets were reviewed to confirm adequate visualization of the optic nerve region and sufficient image quality for ROI delineation. All of the DICOM images were imported into 3D-Slicer (version 4.11.0)^1^ to delineate all segments of the optic nerve on each transverse section. During manual segmentation, the raters were allowed to adjust the display window and level to facilitate visual identification of the optic nerve; however, no windowed or intensity-truncated images were used for radiomics feature extraction. The region of interest (ROI) was independently delineated by two neuroradiologists having more than 10 years of clinical experience. Additionally, 30 randomly selected patients were segmented twice by the neuroradiologists after 1 month to ensure the stability and reproducibility of the intraobserver and interobserver segmentation. The reproducibility of the data was evaluated using the intra-class and inter-class correlation coefficients (ICC). In cases with less distinct margins, delineation relied on overall anatomic continuity and expert judgment; therefore, the reported ICC values should be interpreted as evidence of reproducibility under the study protocol rather than proof of a perfectly unambiguous boundary in every case. The dataset was randomly divided in a 7:3 ratio into a training and a test dataset. All cases in the training dataset were utilized to train the predictive models, while cases in the test dataset were used for independent evaluation of the model’s performance. Medical imaging volumes often exhibit heterogeneous voxel spacing due to variations in scanners or acquisition protocols. This spacing refers to the physical distance between two pixels in an image. Spatial normalization is frequently employed to mitigate the effects of voxel spacing variation. In our experiment, we applied a fixed resolution resampling method to address these issues. All radiomics analyses were performed on the original HU-preserving CT images, and all images were resampled to a standardized voxel size of 1 × 1 × 1 mm. To further minimize potential batch effects arising from heterogeneous CT scanners and acquisition protocols, all extracted radiomics features underwent ComBat harmonization. This normalization ensures the robustness of the model across different imaging platforms.

### 2.5 Radiomics feature extraction

Radiomics feature extraction was performed using the PyRadiomics library in Python. A comprehensive set of features was obtained, encompassing first-order statistics, shape-based descriptors, and textural features derived from matrices such as the gray-level co-occurrence matrix (GLCM), gray-level run length matrix (GLRLM), gray-level size zone matrix (GLSZM), and neighboring gray-tone difference matrix (NGTDM). Furthermore, higher-order features were computed from the aforementioned features using various filters, including wavelet (LLL, LLH, LHL, LHH, HLL, HLH, HHL, HHH), Laplace-Gauss (with sigma values of 1, 2, and 3), square root, logarithm, exponent, gradient transform, 2D local binary pattern (lbp-2D), and 3D local binary pattern (lbp-3D) methods. All the calculation formulas and the pipeline can be found at https://pyradiomics.readthedocs.io/en/latest/. Extracted features subsequently underwent Z-score normalization to standardize their scales.

### 2.6 Radiomics feature selection

A rigorous four-step feature selection was implemented. Initially, features demonstrating poor reproducibility (ICC < 0.9) were excluded. Subsequently, the Mann–Whitney *U* test or *t*-test was applied to identify features significantly associated with outcome (*p* < 0.05). Third, to mitigate multicollinearity, we calculated Pearson’s correlation coefficients among the remaining features; in highly correlated pairs (|*r*| > 0.9), the feature with the stronger univariate association with outcome was retained. The final selection step employed Least Absolute Shrinkage and Selection Operator (LASSO) regression with 10-fold cross-validation on the training set. The optimal tuning parameter (*λ*) was chosen based on the minimum cross-validation error criterion. Features with non-zero coefficients from the LASSO model were retained to construct the radiomics signature, and a per-patient radiomics score was computed as a linear combination of these selected features, weighted by their respective coefficients. The Python scikit-learn package was used for the LASSO regression modelling.

### 2.7 Model construction and validation

We constructed three distinct models. A clinical model was built using independent predictors identified via univariate and subsequent multivariate logistic regression with stepwise selection. A radiomics model was formulated based on the selected radiomics signature. Finally, a combined clinical-radiomics nomogram was developed by integrating the radiomics score and significant clinical variables into a multivariate logistic regression framework. Model performance was assessed by the area under the receiver operating characteristic curve (AUC). Calibration was evaluated using calibration plots and the Hosmer–Lemeshow test. The clinical utility of the nomogram was quantified through decision curve analysis (DCA) across a range of threshold probabilities.

### 2.8 Statistical analysis

Continuous variables were compared using independent *t*-tests, and categorical variables were analyzed with the *χ*^2^ test. Univariate and multivariate logistic regression analyses were conducted with IBM SPSS Statistics (v27.0). Feature extraction leveraged the PyRadiomics (v2.12) package in Python. LASSO regression, ROC analysis, and DCA were implemented in R (v4.2.1) utilizing the ‘glmnet’, ‘pROC’, and ‘rmda’ packages, respectively. A *p*-value below 0.05 was considered statistically significant.

## 3 Results

### 3.1 Clinical characteristics of patients and OCD outcomes

A total of 169 patients were included in the current study, with 118 assigned to the training cohort and 51 to the test cohort. The baseline demographic and clinical characteristics of the two cohorts are shown in Table 1. The average follow-up periods for the training and test cohorts were 56.0 months (ranging from 3 to 133 months) and 60.1 months (ranging from 3 to 131 months), respectively. During the follow-up period, poor outcomes occurred in 49 patients (41.5%) in the training cohort and in 23 patients (45.1%) in the test cohort.

**Table 1:** Demographic and clinical characteristics of patients in the training and test cohort.

Characteristics	Training cohort	Test cohort	*p* value
*n*	118	51	
Age, mean ± SD	39.56 ± 14.57	44.16 ± 15.11	0.064
Follow-up (month), mean ± SD	55.95 ± 36.44	60.12 ± 39.26	0.506
Outcome, *n* (%)			0.109
Poor	49 (41.5%)	23 (45.1%)	
Favorable	69 (58.5%)	28 (54.9%)	
Preoperative VA, *n* (%)			0.633
NLP	74 (62.7%)	30 (58.8%)	
LP	44 (37.3%)	21 (41.2%)	
OCF, *n* (%)			0.797
Yes	65 (55.1%)	27 (52.9%)	
No	53 (44.9%)	24 (47.1%)	
Time to surgery (day), *n* (%)			0.179
3–7	55 (46.6%)	16 (31.4%)	
<3	32 (27.1%)	17 (33.3%)	
>7	31 (26.3%)	18 (35.3%)	
Sex, *n* (%)			0.476
Male	98 (83.1%)	40 (78.4%)	
Female	20 (16.9%)	11 (21.6%)	
Side, *n* (%)			0.728
Right	66 (55.9%)	30 (58.8%)	
Left	52 (44.1%)	21 (41.2%)	
Traumatic factors, *n* (%)			0.251
Traffic accident	69 (58.5%)	38 (74.5%)	
Falling injuries	26 (22%)	6 (11.8%)	
Assault	13 (11%)	4 (7.8%)	
Other	10 (8.5%)	3 (5.9%)	
GCS, median (IQR)	15 (14, 15)	15 (14, 15)	0.732
Surgical approach, *n* (%)			0.041
Pterional	84 (71.2%)	31 (60.8%)	
Supraorbital	20 (16.9%)	6 (11.8%)	
Transnasal	14 (11.9%)	14 (27.5%)	
Postoperative VA, *n* (%)			0.730
LP	40 (33.9%)	19 (37.3%)	
NLP	32 (27.1%)	15 (29.4%)	
HM	16 (13.6%)	8 (15.7%)	
FC	18 (15.3%)	7 (13.7%)	
Standard logarithmic VA	12 (10.2%)	2 (3.9%)	
VA improvement level, mean ± SD	1.11 ± 1.13	0.84 ± 1.03	0.150
Intracranial hematoma, *n* (%)			0.670
No	39 (33.1%)	14 (27.5%)	
Cerebral contusion	39 (33.1%)	19 (37.3%)	
Subdural hemorrhage	13 (11%)	3 (5.9%)	
Combination thereof	9 (7.6%)	4 (7.8%)	
Epidural hemorrhage	18 (15.3%)	11 (21.6%)	

VA: visual acuity; ITON: indirect traumatic optic neuropathy; OCD: optic canal decompression; OCF: optic canal fracture; NLP: no light perception; LP: light perception; HM: hand motion; FC: finger counting; GCS: Glasgow coma scale.

### 3.2 Establishment and performance of the clinical model

**Figure 3:**
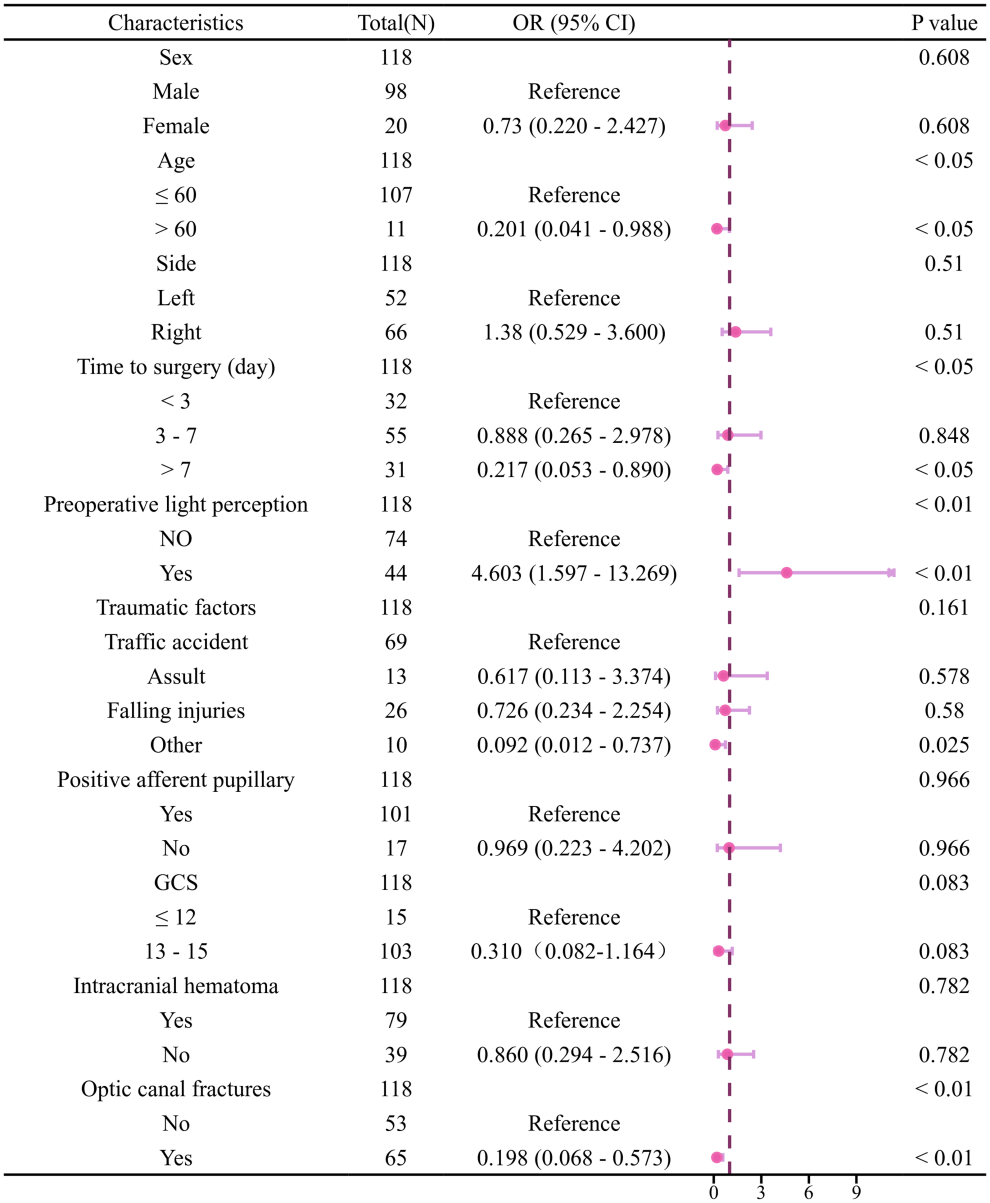
Forest plot of univariate logistic regression analysis for clinical characteristics associated with visual acuity outcome in the training cohort.

**Figure 4:**
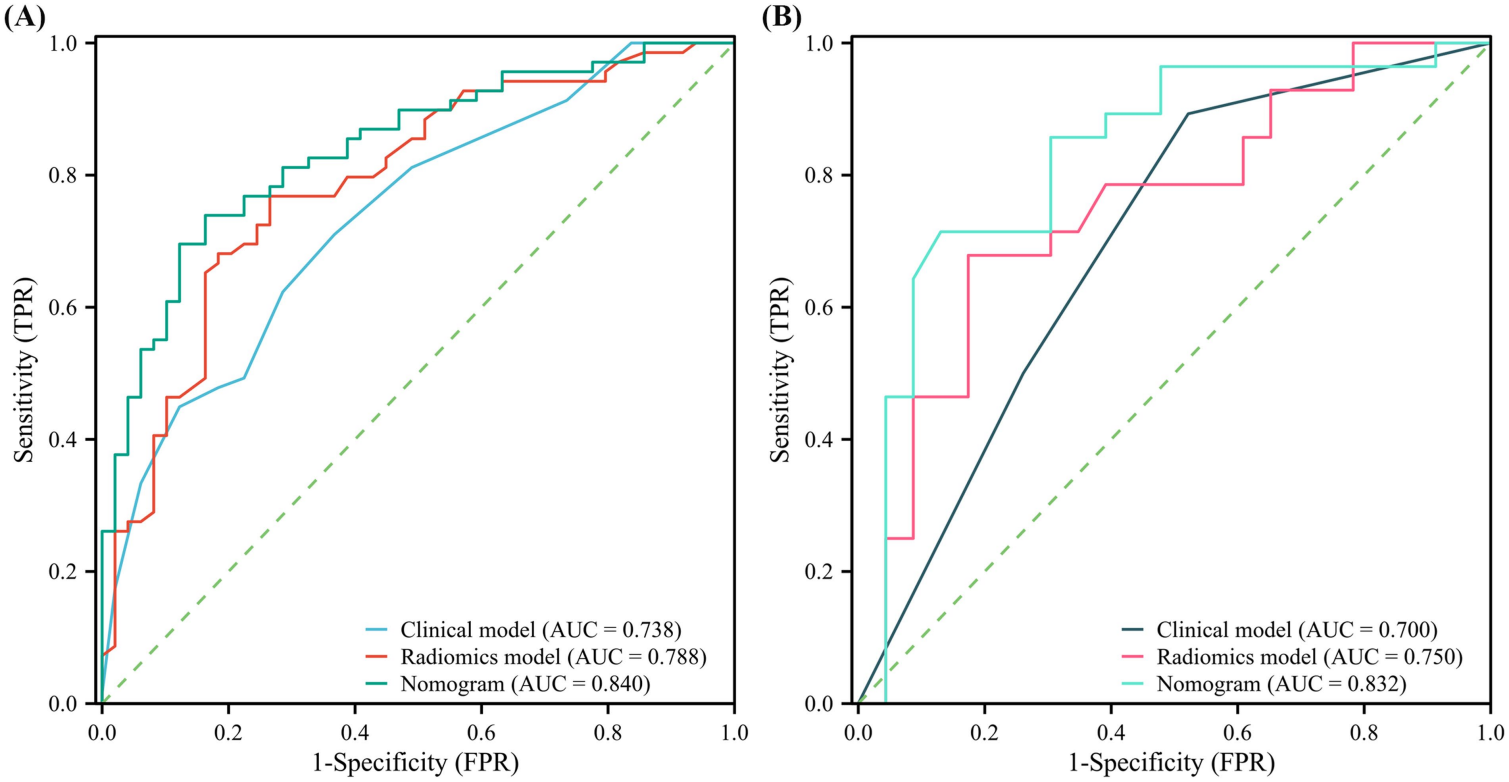
Receiver operating characteristic (ROC) analyses of model performance. ROC curves for the clinical model, radiomics model, and combined nomogram in the **(A)** training and **(B)** test cohorts.

Univariate logistic analysis demonstrated that the initial VA (*p* < 0.01), age (*p* < 0.05), OCF (*p* < 0.01), and time to surgery (p < 0.05) were significantly associated with OCD outcome in the training cohort (Figure 3). Variables with *p* < 0.1 were further analysed using multivariate analysis with the ‘Forward LR’ method. The results indicated that only preoperative NLP, OCF and timing to surgery >7 days were independent risk factors for poor outcomes. Subsequently, a clinical model was established, demonstrating AUCs of 0.738 and 0.700 in the training and test cohorts, respectively (Figure 4). This clinical model effectively distinguished high-risk patients from low-risk patients for poor outcomes in both the training (OR: 15.4; 95% CI: 8.2–28.1; *p* < 0.001) and test (OR: 9.6; 95% CI: 3.6–18.9; *p* < 0.001) cohorts. Low-risk patients exhibited significantly higher rates of VA improvement compared to high-risk patients in the training and test cohorts (73.1% vs. 39.2%, *p* < 0.01, 70.3% vs. 14.3%, *p* < 0.01, respectively).

### 3.3 Radiomics feature extraction and selection

**Figure 5:**
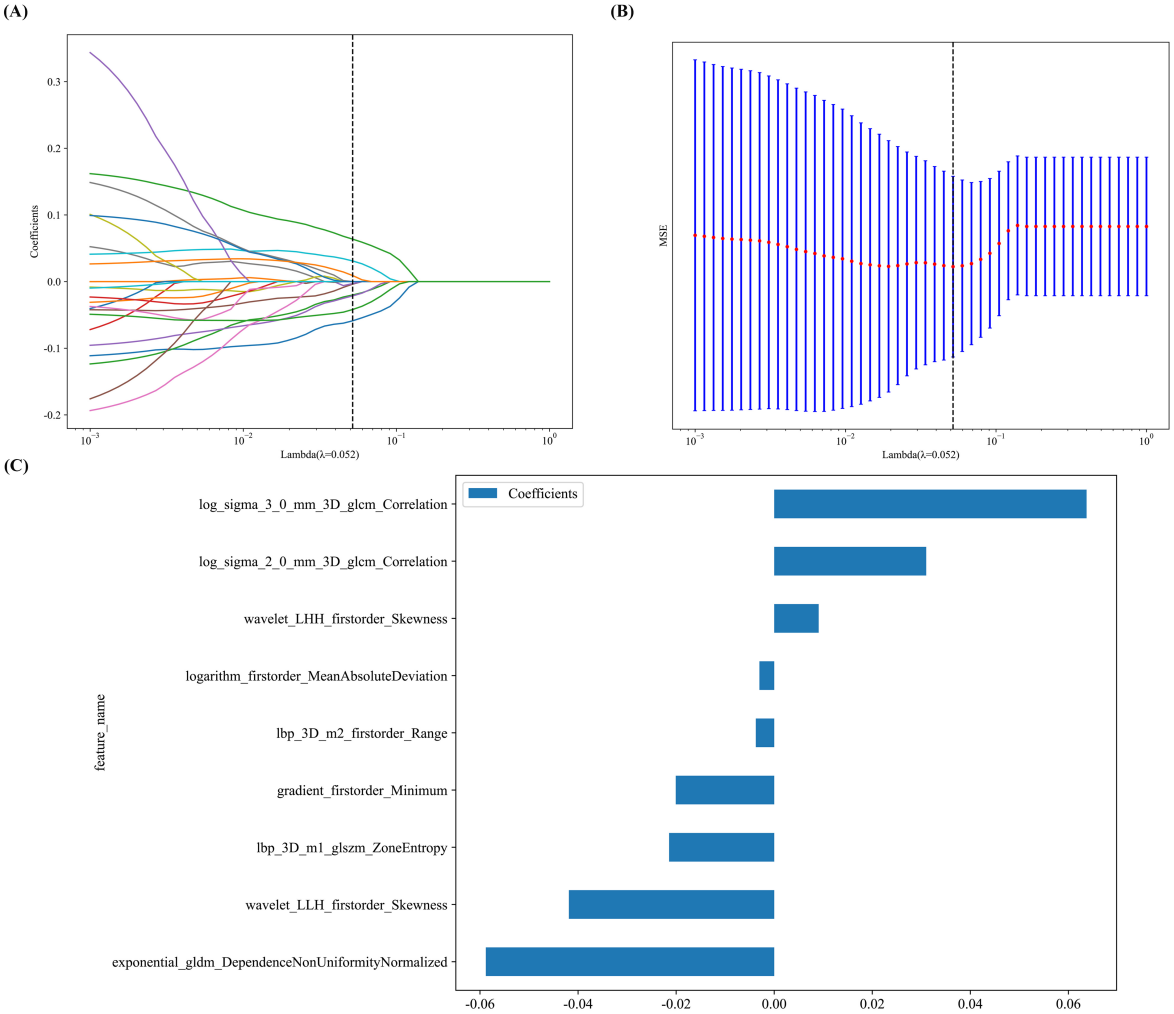
Radiomics feature selection using least absolute shrinkage and selection operator (LASSO) regression. **(A)** Feature coefficient paths across the log(*λ*) sequence. **(B)** MSE of ten-fold cross validation. **(C)** Coefficients of the 9 radiomics features ultimately retained in the signature.

We extracted 1,834 quantitative features from the optic nerve ROIs, including first-order statistics (*n* = 234), shape descriptors (*n* = 14), and textural features (*n* = 1,586). Of these, 1,706 features demonstrated excellent reproducibility (ICC > 0.9). Initial screening via *t*-test/Mann–Whitney *U* test (*p* < 0.05) yielded 37 candidates. Subsequent redundancy reduction using Pearson correlation (|*r*| < 0.9) narrowed the list to 23 features. Finally, LASSO regression with 10-fold cross-validation selected 9 features with non-zero coefficients to form the radiomics signature (Figures 5A–C). Although shape descriptors were included in the initial extraction pipeline, none of them was retained in the final radiomics signature. The mathematical formulation of the signature is provided in Supplementary Appendix E1.

### 3.4 Radiomics model construction and validation

**Figure 6:**
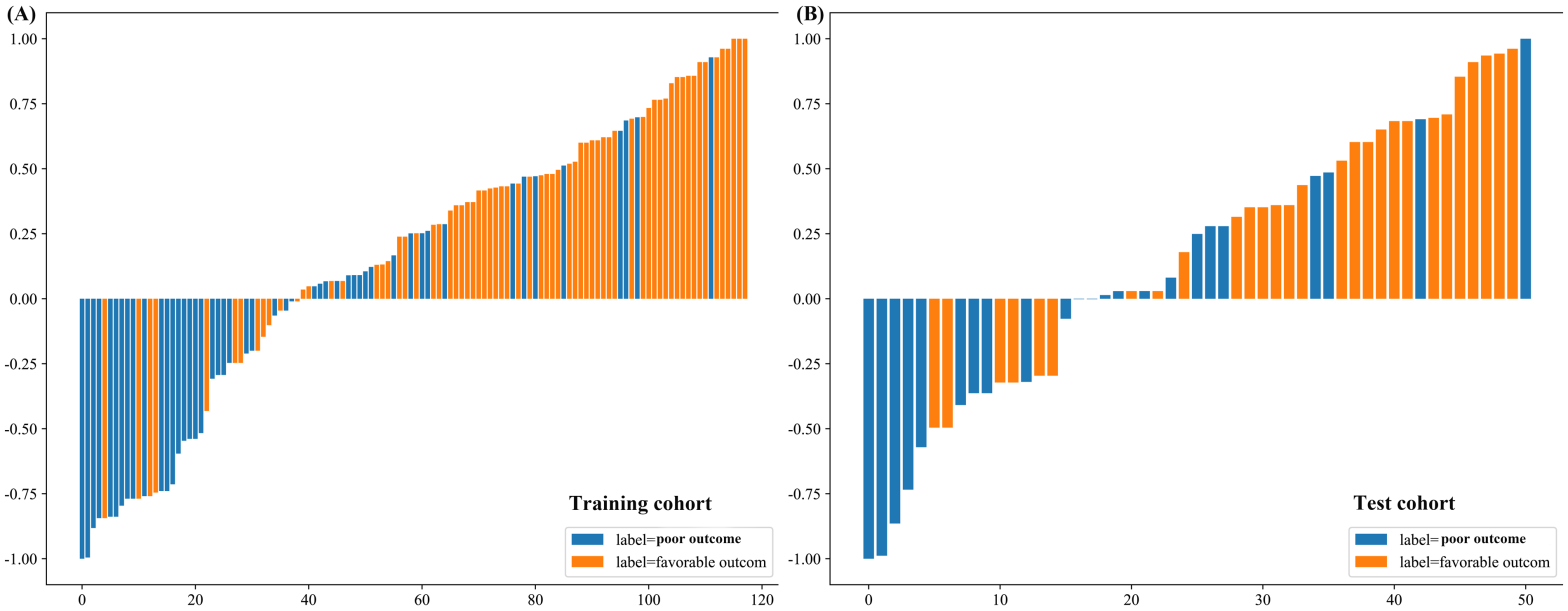
Distribution of radiomics scores. Bar charts showing the distribution of the radiomics score for patients with favorable and poor outcomes in the **(A)** training and **(B)** test cohorts.

The prediction model based on radiomics signatures demonstrated acceptable performance, with AUCs of 0.788 and 0.750 in the training and internal validation cohorts, respectively. The distributions of radiomics scores in the training and test cohorts are presented in Figure 6. In both cohorts, low-risk patients exhibited significantly higher VA improvement rates compared to high-risk patients (79.1% vs. 31.4%, *p* < 0.001; 82.6% vs. 32.1%, *p* < 0.01).

### 3.5 Construction and clinical application of the clinical-radiomics nomogram

**Figure 7:**
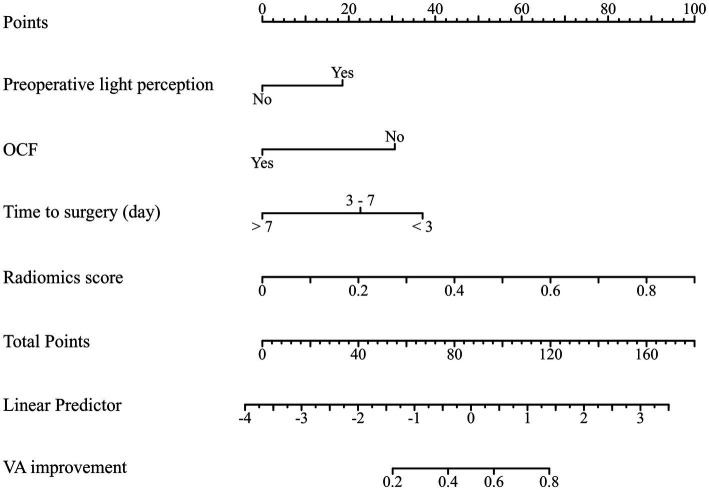
Clinical-radiomics nomogram developed in the training cohort to predict visual acuity improvement.

**Figure 8:**
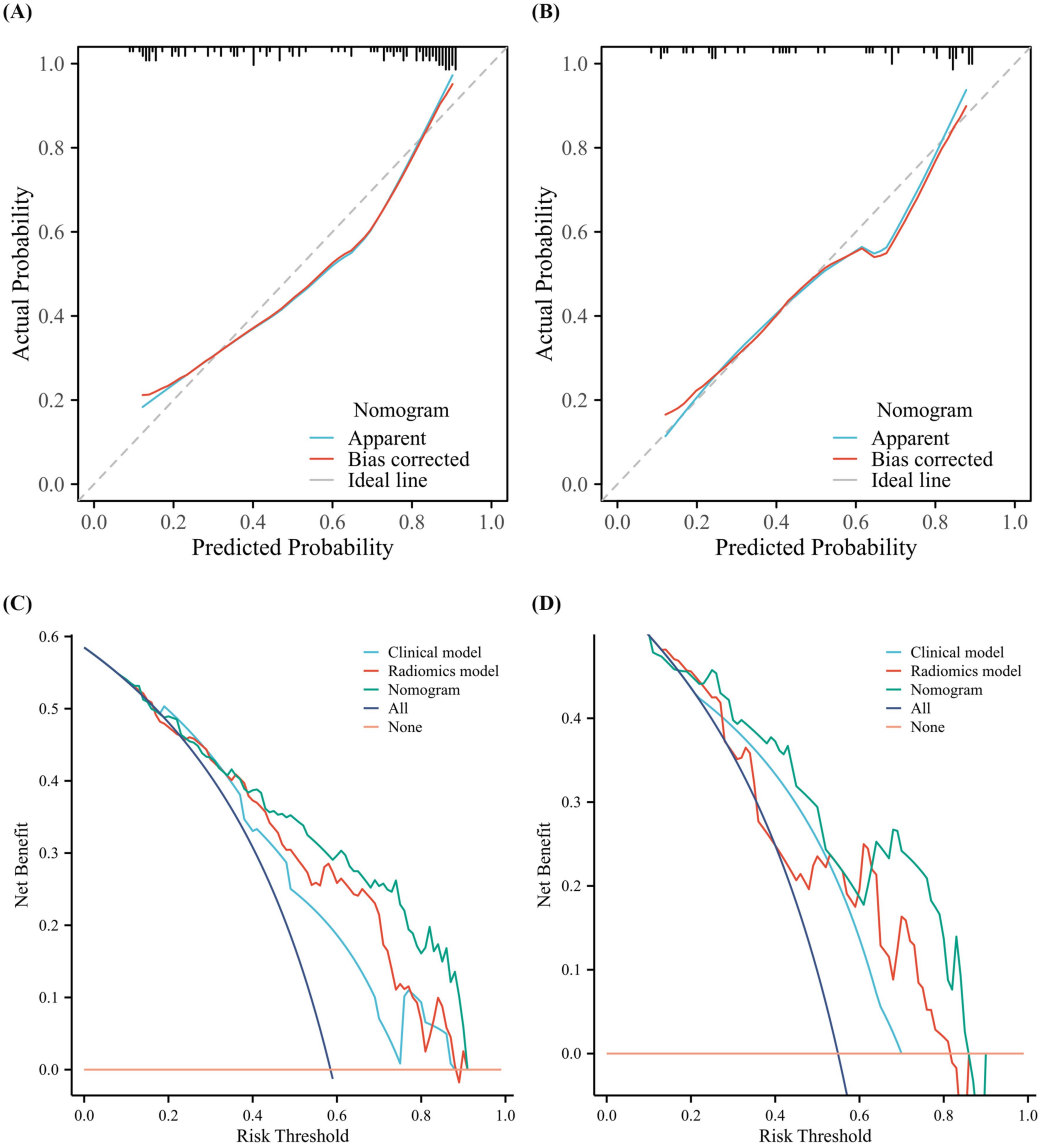
Calibration and decision curve analysis (DCA) for the Clinical-Radiomics Nomogram. Calibration curves for the nomogram in the **(A)** training and **(B)** test cohorts. The 45-degree dotted line represents perfect prediction. Decision curve analysis showing the net benefit of the nomogram across different threshold probabilities in the **(C)** training and **(D)** test cohorts.

The clinical characteristics and radiomics signatures were identified as independent predictors through univariate and multivariate logistic regression analyses, which were subsequently utilized to establish the clinical-radiomics model. This model is presented as a nomogram to provide individualized risk estimates (Figure 7). The AUCs of the nomogram in predicting OCD outcomes were 0.840 and 0.832 in the training and internal test cohorts, respectively. The DeLong test revealed that the nomogram exhibited enhanced predictive performance compared to the clinical model in both the training cohort (*p* = 0.011, DeLong test), and the validation cohort (*p* = 0.020, DeLong test). The sensitivity, specificity, positive predictive value, negative predictive value, and accuracy of all models are shown in Table 2. The clinical-radiomics nomogram successfully stratified patients into high-risk and low-risk categories in both the training (OR: 34.5; 95% CI: 9.5–85.7; *p* < 0.001) and test (OR: 18.3; 95% CI: 7.9–46.8; *p* < 0.001) sets. In the training and test cohorts, low-risk patients demonstrated significantly higher favorable outcome rates than high-risk patients did (87.9 vs. 30.5%, *p* < 0.001; 87.0 vs. 28.6%, *p* < 0.01; respectively). Calibration plots for the clinical-radiomics nomogram demonstrated that the model-predicted VA improvement rates were well calibrated in both the training (*p* = 0.885, Hosmer–Lemeshow test) and test (*p* = 0.626, Hosmer–Lemeshow test) cohorts, as shown in Figures 8A,B. DCA graphically demonstrated that the nomogram provided the largest net benefit across the range of most threshold probabilities compared to other models (Figures 8C,D).

**Table 2:** Predictive performance of the clinical, radiomics, and combined nomogram models for visual acuity improvement.

Model	Cohort	AUC	AUC 95%-CI	Acc	Sen	Spe	PPV	NPV	Youden index
Clinical model	Train	0.738	0.649–0.827	0.710	0.632	0.677	0.731	0.608	0.343
	Test	0.700	0.558–0.841	0.692	0.478	0.706	0.676	0.786	0.371
Radiomics model	Train	0.788	0.705–0.872	0.754	0.768	0.735	0.803	0.692	0.503
	Test	0.750	0.611–0.889	0.745	0.679	0.826	0.826	0.679	0.505
Nomogram	Train	0.840	0.769–0.911	0.739	0.837	0.780	0.864	0.695	0.576
	Test	0.832	0.712–0.952	0.714	0.870	0.784	0.868	0.714	0.584

SEN: sensitivity; SPE: specificity; AUC: receiver operating characteristic curves and the area under the curve; PPV: positive predictive value; NPV: negative predictive value; Acc: accuracy.

## 4 Discussion

This study constructed and internally validated a nomogram for predicting visual acuity (VA) improvement after optic canal decompression (OCD) in individuals with indirect traumatic optic neuropathy (ITON). The model combines three clinical variables with nine radiomics features extracted from preoperative CT scans, showing consistent and strong predictive performance for surgical outcomes. Notably, this integrated nomogram provided better prognostic accuracy and greater potential for clinical application than models based solely on clinical factors or radiomics features. The ability to preoperatively stratify patients into high-risk and low-risk categories for poor VA recovery offers a practical approach to optimizing treatment planning and patient counselling.

While not fatal, ITON typically causes severe monocular visual loss, impairing both the visual field and stereoscopic perception (33). This impairment considerably impacts patients’ professional capabilities and daily life. Management often begins with corticosteroid pulse therapy, but its effect is limited, with only about 55.3% of patients showing VA improvement (7, 34). Consequently, many patients require surgical options for further visual recovery. OCD is the main surgical procedure for these cases, yet its success rate averages only 50%, meaning a significant number of patients undergo surgery without benefit (35–37). This reality highlights the critical importance of carefully evaluating the risks and potential rewards of surgical intervention. In our cohort, which included follow-up data spanning up to 10 years, the VA improvement rate was 57.4%, aligning with the efficacy rates reported in the existing literature.

Preoperatively identifying patients with a low probability of poor outcomes is crucial for improving the overall effectiveness of OCD. Thus, predicting surgical results is vital for creating personalized treatment plans. Several studies have aimed to find clinical and CT features linked to OCD outcomes, identifying factors like preoperative pupillary reflexes, light perception, and injury cause (23). Unfortunately, models based only on these conventional factors often lack sufficient accuracy and perform inconsistently across different patient groups. Our analysis, using univariate and multivariate methods, confirmed that no light perception before surgery, the presence of an optic canal fracture, and a delay to surgery of more than 7 days were independent predictors of poor VA recovery. However, the predictive model built from these clinical factors alone showed only moderate performance. Unlike some previous reports, we did not find a significant link between corticosteroid therapy history, older age, or injury cause with surgical outcomes in our patients. It is also important to note that the choice of surgical approach (e.g., pterional vs. endoscopic) did not influence VA results, implying that the surgical method can be chosen based on the specific fracture pattern and associated injuries without affecting the prognosis. Importantly, having risk factors like NLP or OCF does not automatically disqualify a patient from surgery. Evidence from meta-analyses and randomized trials suggests that surgery can be superior to corticosteroids alone in certain scenarios (21, 38, 39). Therefore, for motivated patients, OCD might still be the preferred option despite the presence of negative predictors.

Radiomics is an advanced method that extracts a large number of quantitative features from medical images, capturing patterns of intensity and spatial heterogeneity that may not be appreciable on conventional visual inspection (40–42). In optic nerve disorders, radiomics has already improved diagnostic precision; for instance, one model combining fundus photos and MRI better distinguished between diabetic retinopathy, glaucoma, and optic neuritis (43). However, its use for outcome prediction in ITON remains relatively unexplored. Because trauma can affect any part of the optic nerve, we contoured the entire nerve on CT scans to capture a comprehensive set of imaging features. This approach yielded nine stable CT-based radiomics features that were associated with OCD outcome. However, the retained higher-order features in this study cannot be uniquely mapped to specific pathological processes such as edema, axonal injury, hemorrhage, or bony impingement. Therefore, the radiomics score should be interpreted as a composite statistical imaging biomarker with prognostic value, rather than as a direct biological surrogate.

When clinical factors were integrated with the radiomics signature, predictive performance improved further. The resulting clinical-radiomics nomogram effectively separated patients into low-risk and high-risk groups in both the training and test cohorts, with markedly different rates of VA improvement between these strata. The difference in outcomes between these groups was striking: patients in the low-risk group had an 87.0% chance of VA improvement, compared to only 28.6% in the high-risk group. Although this supports the potential utility of the nomogram in preoperative risk assessment, we agree that predictive performance alone is insufficient for full clinical translation. The current model should therefore be viewed as a prognostic tool with preliminary clinical utility rather than a fully interpretable stand-alone decision instrument.

Our research has several limitations that should be considered. First, as a retrospective single-center study, it remains subject to selection bias and requires external validation. Nevertheless, the present study provides preliminary evidence that CT-based radiomics may contribute to preoperative risk stratification in ITON. Second, the imaging analysis was based only on CT, and although manual segmentation showed high reproducibility, defining the optic nerve boundary on trauma CT remains inherently challenging; moreover, retrochiasmal visual-pathway injury and subtle retinal pathology could not be fully assessed because dedicated MRI and standardized OCT data were not consistently available. Third, we did not include several clinician-verifiable morphologic imaging metrics, such as minimum optic canal diameter or nerve-to-canal ratio, because these measurements were considered insufficiently robust in the present trauma CT dataset due to the small size of the structures of interest, oblique anatomy, fracture-related distortion, and partial-volume effects. Future studies using dedicated high-resolution imaging or MRI may enable better integration of interpretable morphologic variables with radiomics.

## 5 Conclusion

In summary, our findings indicate that CT-based radiomics features provide significant additional prognostic value beyond standard clinical factors for predicting outcomes of OCD in ITON patients. The clinical-radiomics nomogram may serve as a promising prognostic tool for identifying patients more likely to benefit from surgical intervention. Before widespread clinical adoption can be recommended, further research is necessary to confirm the model’s effectiveness in diverse patient populations and to bridge the gap between this research finding and routine clinical use.

## Data Availability

The original contributions presented in the study are included in the article/Supplementary material, further inquiries can be directed to the corresponding authors.

## Footnotes

1
http://www.slicer.org/
