# Supplementary material for: A CT radiomics nomogram predicts visual acuity improvement in patients with indirect traumatic optic neuropathy following optic canal decompression
Source: Front Neurol. 2026 May 18;17:1819666. doi: 10.3389/fneur.2026.1819666 (PMC13224080; doi:10.3389/fneur.2026.1819666)
Supplement: Supplementary file 1 [file Supplementary_file_1.docx]

Supplementary Material

# Supplementary Data

**Radiomic Signature (Score) Calculation Formula =**

0.5847457627118645 –

0.058808 * exponential_gldm_DependenceNonUniformityNormalized –

0.020072 * gradient_firstorder_Minimum –

0.021426 * lbp_3D_m1_glszm_ZoneEntropy –

0.003745 * lbp_3D_m2_firstorder_Range +

0.031000 * log_sigma_2_0_mm_3D_glcm_Correlation +

0.063705 * log_sigma_3_0_mm_3D_glcm_Correlation –

0.003009 * logarithm_firstorder_MeanAbsoluteDeviation +

0.009071 * wavelet_LHH_firstorder_Skewness –

0.041875 * wavelet_LLH_firstorder_Skewness

.
